# Genetic variation and structure of maize populations from Saoura and Gourara oasis in Algerian Sahara

**DOI:** 10.1186/s12863-018-0655-2

**Published:** 2018-08-01

**Authors:** Miyassa Meriem Aci, Antonio Lupini, Antonio Mauceri, Abdelkader Morsli, Lakhdar Khelifi, Francesco Sunseri

**Affiliations:** 1 LRGB, École Nationale Supérieure Agronomique (ENSA : ES1603), Avenue Pasteur, Hassan Badi, 16200 Algiers, El Harrach Algeria; 2 Dipartimento AGRARIA, Università Mediterranea di Reggio Calabria, Località Feo di Vito snc, 89121 Reggio Calabria, Italy

**Keywords:** *Zea mays* L., Landraces, Genetic resources, Genetic diversity, Phenotypic variation, SSR markers

## Abstract

**Background:**

The ability of maize populations/landraces to tolerate drastically extreme environments over the past four centuries in Algeria leads to characterize these genetic resources for germplasm management as well as the identification of the best landraces useful for genetic improvement. Thus, the aim of the present work was a fingerprinting of an Algerian maize collection (47 landraces) from Saharan oasis by using 24 agro-morphological traits and18 Simple Sequence Repeats to evaluate genetic diversity and population structure.

**Results:**

Phenotypic traits showed large significant variation in which earliness, plant size, ear and kernel features and crop yield appeared the most discriminant traits among landraces by using principal component analysis (PCA). One hundred ninety-seven different alleles were detected with a high mean number of allele per locus (10.9). The selected SSR were highly informative with PIC values > 0.65 as well as an overall genetic diversity (0.47) highlighting a broad genetic variability in the analyzed landraces. Genetic structure analysis revealed a high genetic differentiation among the 47 maize landraces with an overall Fst value (0.33). Cluster analysis for morphological traits as well as for SSR markers grouped the 47 Algerian populations regardless their geographic origin.

**Conclusions:**

Maize from Algerian desert harbors a wide genetic diversity offering a source of novel/unique alleles useful for maize breeding programs to face the ongoing and future major challenge, the climate changes.

**Electronic supplementary material:**

The online version of this article (10.1186/s12863-018-0655-2) contains supplementary material, which is available to authorized users.

## Background

Maize is one of the most diverse crop in the world [1], characterized by a high degree of genetic variability due to an extended selection process before its spread from Central America to the other regions of the world [2, 3].

The introduction of maize in African agro-systems goes back to the sixteenth century, as previously reported [4]. In Algeria, maize cultivation was reported in several regions (Kabylie, Tell and Saharan oasis) by general Duval in 1856 [5], but in the Algerian desert since the sixteenth century [6]. Maize is still appreciated by the Arabo-Berbers of Saharan oasis in South Algeria where several names were attributed to this cereal: *Mastoura, Ktania, Safra, Abidiya, Dora* (in Arabic), *Tifsi* or *Engafouli* (in Tamacheq) [6]. Over this time, maize adapted to the drastic soil and climatic conditions of Algerian desert (dry subtropical climate), where the farmers maintained the useful high genetic diversity through traditional low-pressure selection [7]. More interestingly, crop evolution and maize divergent selection took place and probably still takes place in the traditional farming systems [8]. Therefore, Saharan oasis traditional plant populations or landraces could represent a unique and valuable germplasm.

Although phenotypic variations represent an ideal tool to determine, the agronomic crops performance as well as being inexpensive and comfortable [9], they suffer from low degree of polymorphism and they are affected by environment, thereby determining a limited heritability in germplasm collections [10]. By contrast, DNA-based molecular markers, as microsatellite (Simple Sequence Repeat, SSR), are so far the most used for large scale maize genetic diversity analysis and population structure being highly polymorphic, codominant, abundant in the genome and highly reproducible [11–18]. However, morphological and molecular characterizations are both essential and complementary to study and manage genetic diversity in maize germplasm as well as other crops.

Although maize germplasm from Algerian desert was recently studied by phenotypic and genetic analysis [7, 19], to date the genetic maize pool available in Algeria is not completely evaluated. In particular, Djemel et al. [7] reported a preliminary approach using 10 open-pollinated populations based only on agronomic evaluation, whereas Aci et al. [19] showed genetic analysis of a limited number (15) of Algerian maize populations and therefore not representative of Algerian germplasm. The aim of the present paper was to fingerprint a large collection of Algerian maize landraces (47) from Saharan oasis by both phenotypic traits and molecular markers (18 SSRs) to evaluate genetic diversity and populations structure useful to select genotypes harboring traits of agronomic interest for future breeding programs.

## Results

### Morphological diversity

ANOVA showed a significant block effect for 4 out of 24 traits (DA, KT, 1000 KW and HMC). The seven checks in each replicate significantly differed for many traits except EMG, EV, ASI, ED, EL, HMC and KYP, whereas landraces significantly differed for 13 out of 24 traits including DS, DA, EH, PLH, ERN, ED, CD, RD, KL,KW, EW, 1000 KW and HMC (Additional file 1: Table S4).

The basic statistics of quantitative traits were calculated among all landraces (Table 1). According to the coefficient of variation (CV%), Algerian maize landraces displayed a wide phenotypic variation for ASI (125%), EH (43.88%) PLH (28.50%), KYP (36.15%) and EW (35.43%). By contrast, the lowest variations were observed for kernel features (KL, KW, KT and K%), phenological traits (DA and DS) and ED. The remaining traits showed intermediate levels of variation. At early developmental stages rather all the landraces showed a significant EV, where ENR appeared the most vigorous (Table 1). The earliest landrace was EHA with 69.5 and 68 days after sowing to silking and anthesis, respectively, whereas the latest was KMA (98.52 and 97 days after sowing to silking and anthesis, respectively). A short mean ASI (anthesis -silking interval) was scored (1.22 days), which ranged from 2.52 to 4.47 in BEC and BML, respectively (Table 1), and negative values were detected for seven landraces (DHT, BEC, KTA, DDL, ONA, TIF and AGU). The mean values of PLH and EH was 127.63 and 43.22 cm, respectively; KMA showed the highest (260 cm and 85.70 cm, respectively), while the lowest PLH and EH (79.41 cm and 17.40 cm) were observed in landraces AGU and MNS, respectively (Table 1). NEP ranged from 1.79 (YAK) to 3.19 (AGU). The mean KL was 11.05 mm, the longest value was observed in BSA2 (11.13 mm), while TKR recorded the shortest ones (7.84 mm). BHT and AOR2 showed the highest and lowest values in KW (10.18 and 7.71 mm, respectively), whereas BYA and AOR2 recorded the slimmest and the thickest kernel (KT range from 5.28 to 4.10 mm, respectively). The average KYP was 1.02 Mg ha^−1^ranging from 1.18 (KEK) to7.04 (KMA). However, BTH showed the highest 1000 KW (323.6 g) whereas TBN recorded the lowest value (139.95 g) (Table 1).

**Table 1:** Means and variation of agro-morphological traits in the 47 Algerian maize landraces

Variable				Min		Max		
	Mean	SD	CV %	Value	Landrace	Value	Landrace	Significance
Emergence (EMR, %)	70*.*60	10*.*75	15.23	48*.*73	SAN	92*.*06	KAB	ns
Number of days to reach 50% of the finale % of emergence	12*.*48	1*.*96	15.71	8*.*76	BML	16*.*62	KMA	ns
Early Vigor (EV, 1-9)	5*.*72	0*.*99	17.36	3*.*57	ZDB2	7*.*43	ENR	ns
Days to Silking (DS, days)	80*.*65	6*.*33	7.85	69*.*52	EHA	98*.*53	KMA	***
Days to Anthesis (DA, days)	79*.*43	6*.*57	8.28	68*.*00	EHA	97	KMA	***
Anthesis Silking Interval (ASI, day)	1*.*22	1*.*54	125.98	−2*.*52	BEC	4*.*47	BML	ns
Number of Leaves (NL, #)	4*.*85	0*.*52	10.68	4*.*01	MSN	6*.*71	KMA	ns
Number of Ears Plant^−1^ (NEP, #)	2*.*55	0*.*35	13.89	1*.*80	YAK	3*.*19	AGU	ns
Ear Height (EH, cm)	43*.*22	18*.*96	43.88	17*.*40	MNS	85*.*7	KMA	***
Plant Height (PLH, cm)	127*.*63	36*.*37	28.50	79*.*41	AGU	260*.*01	KMA	***
Ear Row Number (ERN, #)	11*.*11	1*.*69	15.23	8*.*39	BTH	16*.*96	KMA	***
Number of Kernel per Row (NKR, #)	27*.*00	4*.*55	16.86	16*.*21	ENR	40*.*21	KMA	ns
Ear Length (EL, cm)	13*.*41	2*.*27	16.97	8*.*19	TKR	19*.*76	KMA	ns
Ear Diameter (ED, cm)	3*.*51	0*.*34	9.68	2*.*93	DDL	4*.*56	KMA	**
Cob Diameter (CD, cm)	2*.*31	0*.*27	11.65	1*.*88	TKK	3*.*08	GHT	**
Rachis Diameter (RD, cm)	1*.*30	0*.*19	14.53	1*.*01	TKK	1*.*84	KMA	*
Kernel length (KL, cm)	9*.*07	0*.*73	8.05	7*.*84	TKR	11*.*13	BSA2	**
Kernel Width(KW, cm)	8*.*56	0*.*48	5.60	7*.*71	AOR2	10*.*18	BTH	*
Kernel Thickness(KT, cm)	4*.*65	0*.*27	5.89	4*.*10	AOR2	5*.*28	BYA	ns
Kernel proportion(K%)	0*.*83	0*.*05	6.22	0*.*70	SAN	0*.*92	TKK	ns
Weight of 10 ears (EW, Kg)	0*.*78	0*.*28	35.44	0*.*37	TKR	1*.*8	KMA	**
1000 Kernel Weight (1000KW, g)	230*.*97	43*.*07	18.65	139*.*95	TBN	323*.*59	BTH	***
Moisture content at harvest % (HMC)	11*.*50	2*.*34	20.37	6*.*10	TKR	18*.*16	BEC	*
Kernel yield per plot (KYP, Mg ha^−1^)	2*.*82	1*.*02	36.15	1*.*18	KEK	7*.*03	KMA	ns

*EMR%* emergence, *T*_*50*_ *(day)* number days to reach 50% of the final germ, *EV* early vigor, *DS* days to silking, *DA* days to anthesis, *ASI* anthesis silking interval, *NL* number of leaves, *NEP* number of ears plant^− 1^, *EH (cm)* ear height, *PLH (cm)* plant height, *ERN* ear row number, *NKR* number of kernel per row, *EL (cm)* ear length, *ED (cm)*, ear diameter, *CD (cm)* cob diameter, *RD (cm)*, rachis diameter, *KL (cm)* kernel length, *KW (cm)* kernel width, *KT (cm)* kernel thickness, *KP* of kernel, *EW (Kg)* weight of 10 ears, *1000KW* (g)1000 kernel weight, *HMC* moisture content at harvest, *KYP (Mg ha*^*− 1*^*)* Kernel yield per plot

(* = 0.01 < *p* < 0.05; ** = 0.001 < *p* < 0.01; *** = p < 0.001)

The correlations among agro-morphological traits were included in Additional file 2: Table S5. The highest significant positive correlation was between DS and DA (*r* = 0.97) followed by PLH and EH (*r* = 0.92). Interestingly, KYP is highly correlated with PLH and EH, DA and DS, ERN and NKR, while others significant correlations between traits were observed (Additional file 2: Table S5). A limited number of highly significant negative correlations was found, such as KT with DS and DA (*r* = − 0.51 and − 0.46, respectively), ERN and KW (*r* = − 0.39), K% and 1000 KW (*r* = − 0.62) (Additional file 2: Table S5).

**Fig. 1:**
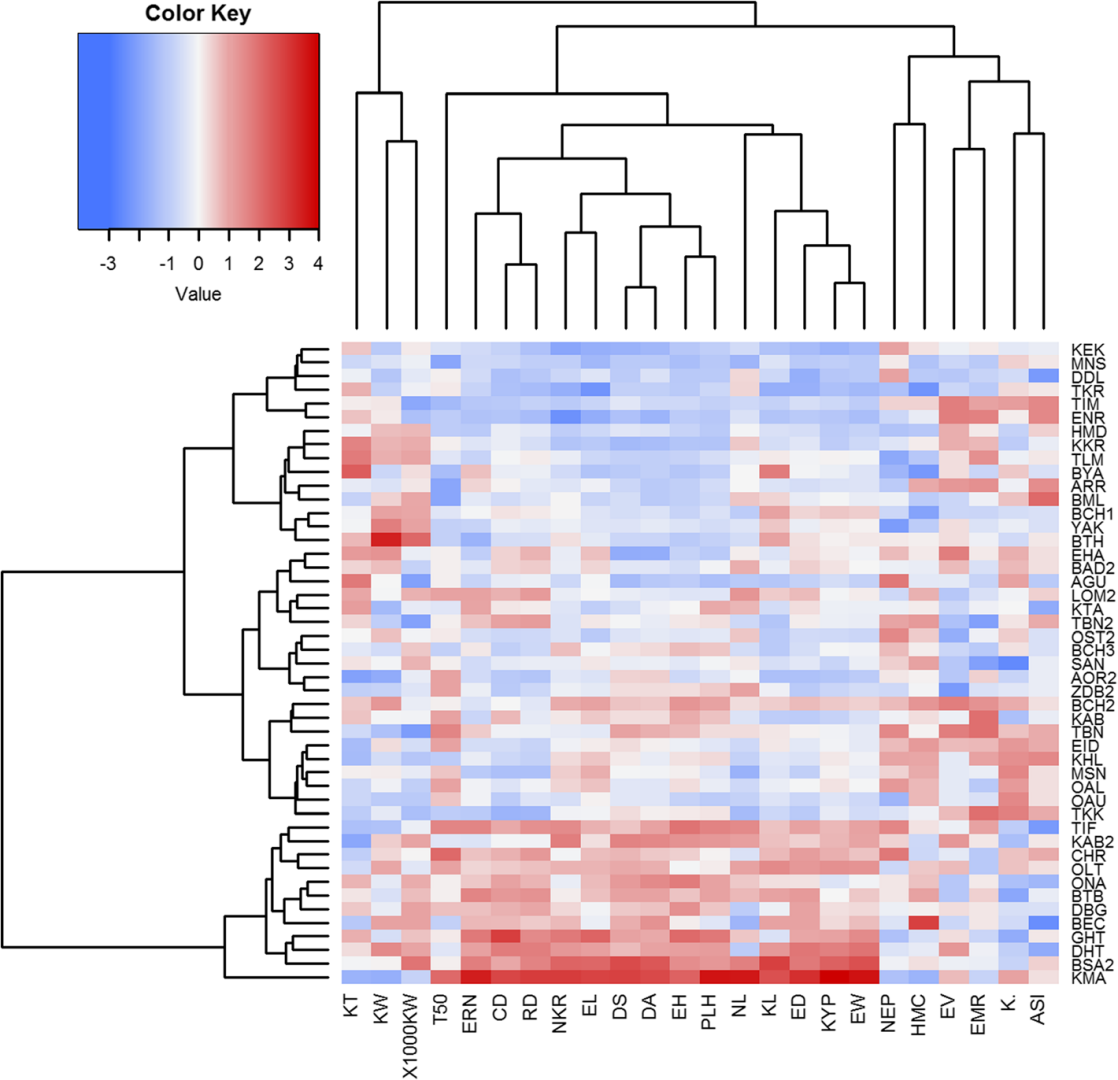
Agro-morphological traits heatmap of the 47 Algerian maize landraces based on Euclidean distances

PCA based on agro-morphological traits displayed three principal components that contributed for 65% to overall phenotypic variability among the Saharan maize landraces (Additional file 3: Table S6). In particular, the PC1 explained 44.29% of total variation, where ear features, earliness, PLH and EH as well as KYP appeared those mainly involved. The PC2accounted for 11.35% of total variation and the traits involved were 1000 KW, kernel sizes and NEP (Additional file 3: Table S6). Furthermore, EV, ASI and KP were traits contributing to the PC3 (9.35% of the total phenotypic variation).The first two PCs (55.64%) were able to distinguish the maize landraces according to their major contributing traits (Additional file 4: Figure S1). Genetic dissimilarity was calculated from agro-morphologic data by cluster analysis based on Euclidean distance and Ward’s method and displayed as heatmap too. Cluster analysis grouped the 47 landraces into three main clusters with distinguished genetic profiles (Fig. 1). The first enclosed the 15 early maturing, short stature and lowest yield landraces. The second cluster grouped 20 landraces with great kernels and high 1000 KW but with the lowest NEP. The third and last cluster was mainly composed of highly vigorous landraces with long ASI and the highest KYP.

### Genetic diversity

SSR summary statistics and genetic indexes among 47Saharan maize landraces are reported in Table 2. All 18 SSR loci, evenly distributed throughout maize genome, were polymorphic. A high number of alleles (197) were detected in bulked DNA samples; allele numbers observed for each locus ranged from 6 (*phi127*) to 20 (*umc1222*) with an average of 10.94 alleles per locus (Table 2). The mean effective number of alleles (Ae), the total gene diversity (H) and Shannon’s information index (I), commonly used to assess population genetic diversity, were also estimated (Ae =1.92, H = 0.46 and *I* = 0.84) exhibiting the highest values for *umc1335* (Ae = 3.00, H = 0.67 and *I* = 1.45) and the lowest for *phi127* (Ae = 1.33, H = 0.25 and *I* = 0.47) (Table 2). Furthermore, the polymorphism information content (PIC) ranged from 0.86 (*umc1335*) to 0.31 (*phi127*) with an whole average value of 0.62 (Table 2). Total genetic differentiation Fst was moderate (0.33), while an excess of homozygotes was observed in our Algerian maize gene pool. A set of 18unique alleles were detected at 14 different SSR loci in the analyzed landraces, and the highest number was recorded in *umc1222* (3 out of 20 alleles) as well as in *umc1403* (2 out of 7). Thirteen landraces out of the 47 revealed unique alleles (Additional file 5: Table S7); the highest number was detected in DHT (3).

**Table 2:** Genetic diversity summary of statistics for 18 SSR loci across the 47 Algerian maize landraces

*Locus*	*Bin*	*Range size*	*A*	*Ae*	*H*	*I*	*PIC*	*Fst*
*umc1222*	*1.01*	*107-190*	*20*	*2.012*	*0.503*	*0.961*	*0.701*	*0.329*
*umc1403*	*1.03*	*113-139*	*7*	*1.715*	*0.417*	*0.730*	*0.558*	*0.305*
*umc1335*	*1.06*	*106-158*	*19*	*3.003*	*0.667*	*1.451*	*0.858*	*0.241*
*umc1165*	*2.01*	*112-157*	*10*	*1.483*	*0.326*	*0.591*	*0.665*	*0.622*
*umc1265*	*2.02*	*93-119*	*8*	*1.811*	*0.448*	*0.726*	*0.735*	*0.427*
*phi127*	*2.08*	*79-153*	*6*	*1.333*	*0.250*	*0.473*	*0.313*	*0.299*
*bnlg1520*	*2.09*	*162-198*	*14*	*1.499*	*0.333*	*0.608*	*0.448*	*0.351*
*phi036*	*3.04*	*57-99*	*15*	*2.160*	*0.537*	*1.070*	*0.701*	*0.27*
*umc1963*	*4.04*	*108-138*	*7*	*1.572*	*0.364*	*0.578*	*0.565*	*0.435*
*umc1329*	*4.06*	*75-109*	*7*	*1.960*	*0.490*	*0.857*	*0.595*	*0.25*
*umc1225*	*5.08*	*80-128*	*20*	*1.957*	*0.489*	*0.978*	*0.656*	*0.317*
*umc1424*	*6.06*	*92-156*	*12*	*2.624*	*0.619*	*1.258*	*0.78*	*0.239*
*bnlg1740*	*6.07*	*93-186*	*10*	*1.801*	*0.445*	*0.788*	*0.529*	*0.215*
*umc1545*	*7*	*43-95*	*8*	*1.923*	*0.480*	*0.803*	*0.747*	*0.381*
*umc1327*	*8.01*	*48-101*	*8*	*2.433*	*0.589*	*1.074*	*0.71*	*0.227*
*umc1984*	*8.03*	*77-109*	*10*	*1.742*	*0.426*	*0.801*	*0.47*	*0.164*
*phi027*	*9.03*	*44-93*	*10*	*1.953*	*0.488*	*0.860*	*0.722*	*0.392*
*phi059*	*10.02*	*139-215*	*6*	*1.541*	*0.351*	*0.550*	*0.438*	*0.404*
*Mean*	*–*	*–*	*10.944*	*1.918*	*0.457*	*0.842*	*0.622*	*0.326*

*A* number of alleles, *Ae* effective number of alleles, *I* Shannon’s Information index [Lewontin (1972)], *PIC* polymorphic information content, *H* total gene diversity, *F*_*ST*_ the inbreeding coefficient within subpopulations relative to the total population (genetic differentiation)

Average of different alleles (Na) was the highest in YAK (4.53) and the lowest in EID (2.63), and gene diversity (H) of a single landrace ranged from 0.38 (EID) to 0.60 (YAK). (Additional file 5: Table S7).

Finally, the analysis of the molecular variance (AMOVA) revealed that genetic diversity within populations contributes to 70% of overall genetic diversity (Additional file 6: Table S8).

### Cluster analysis and genetic structure

**Fig. 2:**
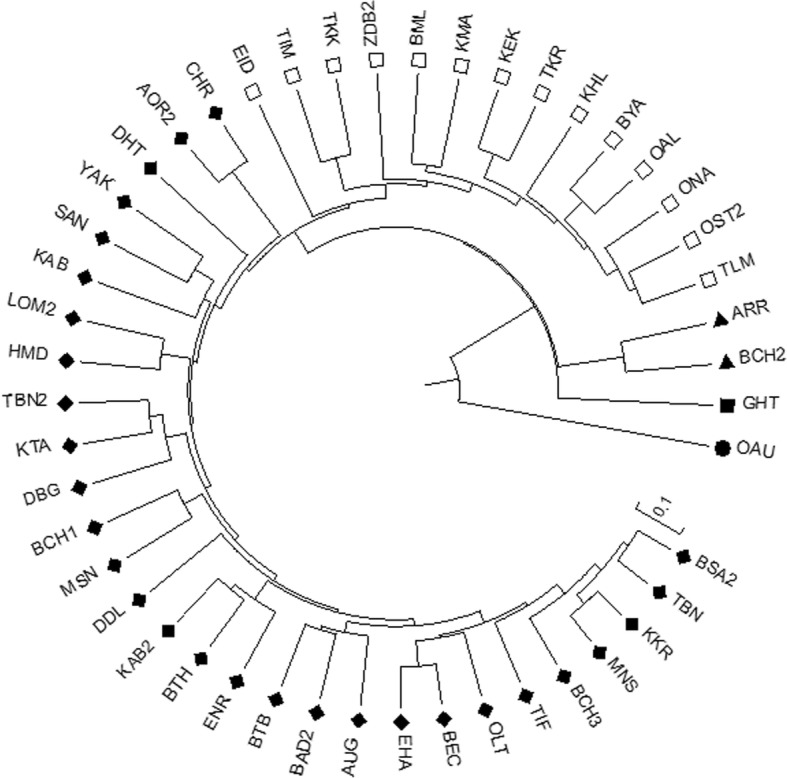
Dendrogram of the 47 Algerian maize landraces based on SSR data according to UPGMA method. Different symbols indicate clusters (♦ = A, □ = B, ▲ = C, ■ = D, ● = E)

Cluster analysis based on SSR data was performed using the genetic distances of Nei and Li [20] and UPGMA algorithm (Fig. 2). Forty-seven landraces were classified in three large clusters (from A to C) and two landraces as out-groups. In particular, cluster A enclosed the highest number of landraces (29), clusters B and C encompassed 14 and 2 landraces, respectively, while the landraces GHT and OAU were the out groups. The highest genetic distance was recorded between cluster C and A, while the lowest was observed between clusters A and B and the landraces OAU and GHT. The average gene diversity was the highest within cluster A and it was higher than total genetic diversity (Additional file 7: Table S9).

The correlation coefficient (*r* = − 0.15, *P* < 0.001) for the two clustering matrices tasted by Mantel’s test revealed a highly significant but negative correlation between the agro-morphological and SSR information.

**Fig. 3:**
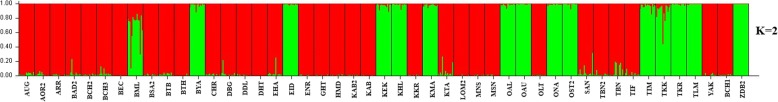
Genetic structure of the 47 maize landraces as inferred by STRUCTURE based on 18 SSR data set

The differences among landraces were further highlighted by STRUCTURE analysis. The optimum number of genetic groups (K) within our collection was determined K = 2 based on ΔK peaks (Fig. 3). Considering the admixture coefficient (Q) ≥ 0.9 as the assignment probability of each population to a group, it was possible to assign 32 and 13 populations to group 1 (red) and 2 (green), respectively (Fig. 3). Two populations, BML and TKK, exhibited Q values of 0.686 and 0.88, respectively, thus showing an admixture genetic structure. Finally, allele frequency divergence between populations was 0.0479, while mean genetic distance within population was 0.6575 and 0.6012 for group 1 and 2, respectively.

## Discussion

Landraces represent an odd genetic resource for crops with a high genetic variation useful for modern breeding programs [17]. Approximately only 5% of maize genetic diversity is reported to be in commercial use [21], while the remaining germplasm have been neglected due to undesirable traits, such as low yield. In maize, landraces are low yielding varieties, but their large genetic diversity ensure crop adaptation to different stresses appearing adequate to meet the ongoing and future major challenge, the climate changes [22].

Therefore, the conservation of landraces as genetic resources and the management of such gene pools are important by ensuring suitable agronomic and genetic knowledge [23]. In the present study, we approached a genetic diversity assessment of 47 maize landraces from Saoura and Gourara Oasis (Algeria) using both agro-morphological and molecular data.

### Morphological diversity

Algerian maize is a promising source of favorable alleles for stress tolerance, but it has been neglected until recently [7], thus the main purpose of the current research was to overview the agro-morphological potential of Saharan maize landraces based on quantitative traits. The estimation of variance revealed significant variability among landraces, suggesting a high phenotypic diversity within the Algerian maize collection. The maize populations varied greatly for plant size, ASI, kernel yield, and 10 ears weight. KMA was the latest, the tallest and the most productive landrace exhibiting the highest grain yield. Indeed, the late maturing maize landraces characterized by short ASI were also the most productive [24] At the phenological stages, all the landraces showed a normal flowering behavior (ASI = 1.22 days) except DHT, BEC, KTA, DDL, ONA, TIF and AGU which showed negative ASI values as a consequence of silk emergence prior to pollen emission. This feature could be a beneficial trait under drought conditions [25] causing delay in silk emergence, increasing the synchronicity between male and female flowering as well as resulting into higher grain yield. In this respect, negative and short ASI as secondary trait employed for breeding programs for drought tolerance was previously reported [26] Morphological traits were subjected to the environment influence as well as natural and human selection [27]. The high variability among flowering behavior (days to silking and anthesis as well as ASI) and crop yield, which are related to maize adaptation to the environment [28], suggested that Saharan landraces were adapted to a large range of environments supported also by alternative agronomic practices in different growing maize regions. Thus, our results are in agreement with a previous report on Algerian maize landraces, which pointed out that even though the landraces originated from subtropical regions, they showed adaptation to both sub-humid and dry continental environments [7]. Significant correlations among agro-morphological traits were identified, representing a useful tool for directing breeding programs [29]. However, environment plays also a pivotal role, affecting concurrently the traits in the same or opposite direction [30]. Such forceful correlation between agro-morphological traits in maize was already reported [15, 31].

The principal component analysis (PCA) was performed to cluster landraces based on discriminant morphologic traits. According to Clifford and Stephenson [32], the first three principal components included the overall phenotypic variation, taken into account discriminant traits able to distinguish the landraces. Among them, plant size, days to flowering, yield, ear as well as kernel features, and 1000 kernel weight were highly significant for clustering maize landraces. Our results are also in agreement with Gouesnard et al. [33], Beyene et al. [34] and Hartings et al. [35], which reported flowering, plant and ear size as the most important traits for grouping French, Ethiopian and Italian landraces, respectively. Further, Sharma et al. [36] also reported that kernel yield and 1000 kernel weight as highly informative traits in maize differentiations. CA as well as PCA analysis were not able to distinguish maize populations from the same geographic origin being dispersed in different clusters. Similar results were already reported by Hartings et al. [35] and Sharma et al. [36], which observed a lack of relationship between clustering and landraces geographic origin. Finally, it is noteworthy that landraces from dry, of poor soil and highly radiated environments origin showed adaptation traits as shortness and early flowering, considered important sources of gene diversity for developing drought tolerant genotypes [31, 34].

### Genetic diversity

The first step for crop improvement strategies consist on the assessment of genetic variation and the relationships among landraces/varieties/populations. SSR markers represent an efficient tool in providing direct and reliable information for a helpful management and conservation of germplasm collections. The genetic diversity of 47 Algerian maize landraces was analyzed in bulk fingerprinting method, leading to detect high levels of polymorphism (10.94 alleles per locus). Higher results were reported by Wasala and Prasanna [37], which found 13.1 alleles per locus (across 42 SSR loci) studying 48 Indian landraces. A less number of alleles per locus compared to our study were detected in maize landraces from Switzerland, Ghana and Turkey with 8, 7.3 and 6.21 alleles per locus, respectively [15, 38, 39]. In a previous report, 5.8 alleles per locus were detected with a 6% polyacrylamide gel electrophoresis using the same SSR set of the present work [19]. The present study strengthened a wide genetic variation in our Algerian maize landraces from Sahara. As expected, we proved the higher efficiency of fluorescent dye labeled SSR markers, with a higher allele resolution and detection using DNA-sequencer, compared to the method adopted by Aci et al. [19]. The PIC average value (0.62) revealed an allelic variation in SSR loci higher than in the US germplasm (0.59), Swiss (0.52) and Ghana (0.50) maize landraces [11, 38, 39]. PIC is considered as the better parameter for measuring genetic diversity than the number of alleles, taking into account the relative frequencies of each allele [40]. This supports the informativeness of the chosen SSR to assess the Algerian maize genetic diversity.

Almost 50% of the SSR used in our study was highly informative (PIC > 0.65) and the overall gene diversity (0.46) was higher compared to the previous report on 15 Algerian landraces (0.40) [19] and comparable to those found in Ghanaian landraces [39] and Spanish populations [41].

Regardless the abundance of SSR in maize genome and their high polymorphism [12, 42], the dinucleotide motif SSR were reported to overestimate the genetic diversity and the number of alleles per locus [43], because of their higher mutation rate compared to oligonucleotide motif SSR [44]. In agreement, 7 out of 9 most informative SSR are dinucleotide repeats (*umc1222* and *umc1335*), showing a high number of alleles (20 and 19, respectively). Furthermore, plant populations harboring unique alleles, due to SSR high mutation rate [45], but also to natural selection of specific alleles along the adaptation to environments [46], are reported to be potential for crop improvement as source of new alleles [47]. It is noteworthy that 18 unique alleles were identified in our maize landraces (almost 10% of total), confirming this percentage of unique alleles previously detected by Aci et al. [19]. Choukan et al. [48] observed 22% of unique alleles on 36 maize inbred lines from Iranby using 42 SSR, while Wasala and Prasanna [37] led to the identification of 31% unique alleles. The present study indicated a moderate differentiation among Saharan maize landraces related to such specific allele fixation in the core set. The total genetic differentiation (Fst = 0.33) was closed to that reveled in the first report across Algerian maize landraces (Fst = 0.31) [19]. Similar Fst values (ranged from 0.33 to 0.35) were previously reported in maize landraces from India, Switzerland and Northern America [38, 45, 49].

The population structure based on the Bayesian admixture-model is in agreement with PCA and cluster analyses as well as the geographic origin of landraces. This result appears in agree with previous studies [42, 50], which reported a lack of clustering based on phenotypes, environmental adaptation, grain color or type, and maturity.

Even if maize is an allogamous plant species, in agreement with our results, a higher genetic variation within population than among them was yet described in other studies [51]. Da Silva et al. [51] considered that SSR loci revealed higher heterozygosity within population, which produced low differentiation among them. As a result, a wide variation is engendered useful for crop improvement [52]. Cluster analysis revealed a significant genetic diversity within and among landraces at both phenotypic and genetic levels, but failed to cluster the landraces according to their geographical origin as previously observed by Cömertpay et al. [15] and Noldin et al. [53].

A negative correlation between morphological and genetic distance matrices was revealed by the Mantel test, in agreement with previous report on ryegrass varieties by Roldan-Ruiz et al. [54]. This suggests that phenotypic and genetic variances are due to independent genome regions [55], being molecular markers selectively neutral and not always mirrored the diversity of expressed traits [56]. These results justify the use of both molecular and phenotypic markers, resulting complementary for assessing maize genetic diversity.

## Conclusions

The Algerian maize landraces hold a significant genetic diversity suggesting their potential adaptation to the extreme Sahara environment, according to genetic parameters and the clustering based on both molecular and agro-morphological markers. Four centuries of growing in the Sahara could lead to a widening of gene pool diversity in maize germplasm, due to genetic recombination, mutations, natural and human selection, as stated for plant populations by Hartl and Clark [57]. Natural selection could gathered favorable genes for tolerance to abiotic stress. Noteworthy, in the present study the best promising tolerant landraces to abiotic stress were identified with the aim to be included in breeding programs for the development of novel water use efficient and drought tolerant maize genotypes.

## Methods

### Plant material and agro-morphological descriptors

The maize germplasm used in this study consisted of forty-seven populations/landraces (a landrace of out-pollinated plant species can be defined “population”) collected during 2009 and 2010 in different Oasis of South-Western Algeria (from 27°52′ to 31°37’ N latitude and 0°17′ to 2°13’ W longitude). In particular, six and thirty-eight populations/landraces were collected from Bechar and Adrar oasis, respectively; whereas three populations/landraces were collected from Gherdaia (1) and Saida (2) (see Additional file 8: Table S1, also for the acronym of each population/landrace).

The trial was carried out at the experimental station of the National School of Agronomy of Algiers (36° 47’ N, 2° 03′E altitude 32 m) in the sub humid North of Algeria during the spring-2013 under field conditions. The experimental design was an Augmented design with three blocks [58] and seven checks of which 2 maize populations from Iowa State University, USA (BS17 and BSL(S)C6), one population from University of Guelph, Canada (Longfellow), and 4 from Spain (Norteño, Rastrojero and Tremesino populations from the Spanish Ministry of Agriculture, and Tuy from Misiòn Biològica de Galicia, Pontevedra) were repeated in each block.

Each experimental plot consisted of one row, 30 hills per row and one grain per hill. Rows were spaced 0.70 m apart and hills were 0.20 m. The data were recorded on ten random plants for twenty-four agro-morphological traits using the standard descriptors suggested by the International Board for Plant Genetic Resources [59] (Additional file 9: Table S2). They include plant emergence (EMG,%), number of days to reach 50% of field germination (T50, days), early vigor (EV), days to silking (DS, days), days to anthesis (DA, days), anthesis-silking interval (ASI, days), number of leaves (NL) and ears per plant (NEP), plant (PLH, cm)and ear height (EH, cm), ear row number (ERN), number of kernels per row (NKR), ear length (EL, cm) and diameter (ED, cm), cob (CD, cm) and rachis diameter (RD, cm), kernel length (KL, cm), width (KW, cm) and thickness (KT, cm), kernel proportion (K%), weight of 10 ears (EW, kg), 1000 kernel weight (1000 KW, g), moisture content at harvest (HMC, %), and kernel yield per plot (KYP, Mgha^− 1^).

### DNA extraction

Maize seeds of each landrace were surface sterilized for 20 min in 20% (*v*/v) sodium hypochlorite solution, rinsed with deionized water and transferred for germination in Petri dish (diameter 9 cm), on filter paper with 0.5 mM CaSO_4_ at 26 °C in dark condition for one week.

The DNA pooled-sampling strategy was employed [60, 61] and the genomic DNA was isolated from 15 seedlings [37] for each population using DNeasy Plant Mini Kit (Qiagen, Milano, Italy), according to manufacturer’s protocol and its quality and quantification was assayed by BioPhotometer D30 (Eppendorf, Hamburg, Germany).

### SSR analysis

Eighteen SSR loci were employed for genotyping the forty-seven landraces [19] (http://www.maizegdb.org/data_center/ssr) (Additional file 10: Table S3). The PCR amplification was carried out with a Thermal Cycler 2720 (Applied Biosystem, Thermo Fisher Scientific Inc.) using specific temperature of annealing (Ta) for each primers pair. The reaction was carried out in 20 μl volume containing 20 ng DNA for each bulk, 1 U Taq DNA polymerase (Thermo Fisher Scientific Inc.), 0.32 μM reverse primer, 0.16 μM forward and 0.16 μM fluorescence (FAM) labeled universal primer M13 (− 21) as reported by [62]. PCR was programmed at 95 °C (5 min), 30 cycles 94 °C (30 s), Ta (45 s) and 72 °C (45 s) followed by 8 cycles 94 °C (30 s), 53 °C (45 s) and 72 °C (45 s), and final extension at 72 °C for 10 min. PCR products were separated by capillary electrophoresis and genotyped with an ABI PRISM 3500 Genetic Analyzer (Applied Biosystem). Allelic data were exported and selected from sequencer using Gene Mapper v.5 software (Applied Biosystem, Thermo Fisher Scientific Inc). Finally, to filter raw data, allele calling and estimate allele frequencies for each bulk and SSR locus the “FreqsR” software was used. In addition, the conversion of allele frequencies into allele sizes in individuals was carried out by “F-to-L” software [12, 16]. Both software were run on R platform (R Development Core Team 2008).

### Statistical analysis

An augmented design was adopted to analyze the landraces for morphological traits [58]. Means of 47 landraces were adjusted by blocking effects of the replicated checks using ACBD-R software released by CYMMYT (International Maize and Wheat Improvement Center). Data were analyzed to find significant differences among landraces for each trait. Standardized means to remove the effects of different scales of measurements was adopted in the Principal Component Analysis (PCA) and Cluster Analysis (CA) As suggested by Clifford and Stephenson [32] and Guei et al. [63], the first three components were used for characterizing and differentiating the landraces. For cluster analysis, data were analyzed to determine Euclidean distance based on paired group method to determine dissimilar groups among landraces. Analyses were performed using R software (R Development Core Team 2008).

To determine genetic parameters such as number of alleles (A), average of different alleles (Na), effective number of allele (e), Nei’s gene diversity index (H), Shannon diversity index (I), forboth SSR loci and population GenAlex software version 6.3 was utilized [64]. Private alleles were determinate using GDA software (http://hydrodictyon.eeb.uconn.edu/people/plewis/downloads/gda-1.1.win32.zip), whereas the Polymorphic information content (PIC) for each SSR locus was also estimated using Cervus v. 3.0.7 software (Copyright Tristan Marshal, Field Genetic, Ltd). The Analysis of Molecular Variance (AMOVA) was also performed to analyze genetic variation among and within individuals [65] by Arlequin software (http://cmpg.unibe.ch/sofware/arlequin35) testing Fst by 9999 random permutations. A dendrogram to define differences among populations was constructed based on Nei and Li [20] pair-wise distances matrix and the unweighted pair group method of arithmetic clustering algorithm (UPGMA) [66] using MEGA v. 6 software [67].

In addition, model-based (Bayesian) clustering was performed to evaluate genetic relationship among individuals and population structure by using Software package STRUCTURE [68]. The program was set up and run as reported in Mercati et al. [69]. Then, the criterion (ΔK) of Evanno et al. [70] was used to determine the most probable K value, in order to compensate for overestimation of subgroup number by STRUCTURE. Finally, as reported by Wang et al. [71], lines with membership probabilities ≥0.90 were assigned to the corresponding subgroups and lines with membership < 0.90 were assigned to a mixed subgroup.

## Additional files

Additional file 1: **Table S4.** Augmented ANOVA (mean squares) for 24 agro-morphological traits in 47 Algerian maize landraces and 7 checks. (DOCX 17 kb)

Additional file 2: **Table S5.** Correlation matrix of 24 agro-morphological traits used to characterize 47 Algerian maize landraces. (XLSX 16 kb)

Additional file 3: **Table S6.** Eigenvalues, variances and coefficients associated with first three principal components. (DOCX 15 kb)

Additional file 4: **Figure S1.** Principal component analysis of the 47 Algerian maize landraces based on 24 agro-morphological traits. (JPEG 152 kb)

Additional file 5: **Table S7.** Landraces summary statistics based on SSR analysis. (DOCX 14 kb)

Additional file 6: **Table S8.** Analysis of Molecular Variance. (DOCX 12 kb)

Additional file 7: **Table S9.** Partition of the gene diversity among clusters. (DOCX 12 kb)

Additional file 8: **Table S1.** List of the 47 Algerian maize landraces. (DOCX 22 kb)

Additional file 9: **Table S2.** Agro-morphological traits recorded in the 47 Algerian maize landraces. (DOCX 15 kb)

Additional file 10: **Table S3.** Primers used to detect SSR marker. (DOCX 14 kb)

## Abbreviations

AMOVA: Analysis of Molecular Variance
CIMMYT/IBPGR: International Board for Plant Genetic Resources
Fst: inbreeding coefficient within subpopulations relative to the total population (genetic differentiation)
H: Nei’s gene diversity index
PCA: principal component analysis
PCR: Polymerase chain reaction
PIC: Polymorphic information content
SSR: Simple sequence repeat
UPGMA: unweighted pair group method using arithmetic averages
