# Supplementary material for: Genetic variation and structure of maize populations from Saoura and Gourara oasis in Algerian Sahara
Source: BMC Genet. 2018 Aug 1;19:51. doi: 10.1186/s12863-018-0655-2 (PMC6090932; doi:10.1186/s12863-018-0655-2)
Supplement: Supplementary file 1 — Table S4. Augmented ANOVA (mean squares) for 24 agro-morphological traits in 47 Algerian maize landraces and 7 checks. (DOCX 17 kb) [file 12863_2018_655_MOESM1_ESM.docx]

**Table S4.** Augmented ANOVA (mean squares) for 24 agro-morphological traits in 47 Algerian maize landraces and 7 checks

| Effect | **df** | **EMR** | **T_50_** | **EV** | **DS** | **DA** | **ASI** |
| --- | --- | --- | --- | --- | --- | --- | --- |
| *Blocks* | 2 | 42.56 | 7.57 | 4.48 | 8.14 | 19.13* | 4.58 |
| *Entries* | 53 | 161.49* | 8.84** | 0.83 | 73.51*** | 71.07*** | 2.47 |
| *Checks (C)* | 6 | 55.20 | 9.87** | 1 | 146.98*** | 160.44*** | 0.53 |
| *Landraces(L)* | 46 | 115.61 | 3.84 | 0.98 | 40.09*** | 43.23*** | 2.36 |
| *C vs L* | 1 | 2909.97*** | 232.8*** | 7.11* | 1169.73*** | 815.72*** | 19** |
| *Error* | 12 | 59.171 | 1.99 | 1.23 | 5.55 | 3.397 | 1.37 |
|  |  |  |  |  |  |  |  |
|  | **df** | **NLP** | **NEP** | **EH** | **PLH** | **ERN** | **NKR** |
| *Blocks* | 2 | 0.34 | 0.15 | 140.68 | 651.4813 | 0.165 | 0.147 |
| *Entries* | 53 | 0.48** | 0.14 | 515.71*** | 4063.115*** | 4.288*** | 36.305** |
| *Checks (C)* | 6 | 1.58*** | 0.21* | 595.9*** | 858.606*** | 15.819*** | 80.844*** |
| *Landraces(L)* | 46 | 0.26 | 0.12 | 359.58*** | 1322.966*** | 2.864*** | 20.73 |
| *C vs L* | 1 | 3.85*** | 0.46* | 7216.55*** | 149337*** | 0.593 | 485.374*** |
| *Error* | 12 | 0.12 | 0.065 | 48.09 | 181.7641 | 0.431 | 9.395 |
|  |  |  |  |  |  |  |  |
|  | **df** | **EL** | **ED** | **CD** | **RD** | **KL** | **KW** |
| *Blocks* | 2 | 1.77 | 0.0076 | 0.0097 | 0.00092 | 0.0267 | 0.0104 |
| *Entries* | 53 | 13.21 | 0.148** | 0.093*** | 0.0565** | 0.816** | 0.954*** |
| *Checks (C)* | 6 | 28.57 | 0.054 | 0.132*** | 0.083** | 1.938*** | 3.65*** |
| *Landraces(L)* | 46 | 5.17 | 0.115** | 0.072** | 0.035* | 0.532** | 0.229* |
| *C vs L* | 1 | 290.67** | 2.25*** | 0.831*** | 0.859*** | 7.158*** | 18.06*** |
| *Error* | 12 | 15.98 | 0.026 | 0.0143 | 0.013 | 0.173 | 0.082 |
|  |  |  |  |  |  |  |  |
|  | **df** | **KT** | **K%** | **EW** | **1000KW** | **HMC** | **KYP** |
| *Blocks* | 2 | 0.200* | 0.0198 | 0.0036 | 14364.74*** | 224.7812*** | 1.692 |
| *Entries* | 53 | 0.139* | 0.0378** | 0.075** | 12168.39*** | 1625.44*** | 21.921*** |
| *Checks (C)* | 6 | 0.371** | 0.095*** | 0.0606* | 63.5*** | 5.321 | 5.322 |
| *Landraces(L)* | 46 | 0.075 | 0.0026 | 0.0771** | 1854.788*** | 5.485* | 1.0374 |
| *C vs L* | 1 | 1.700*** | 1.309*** | 0.0915* | 559223.2*** | 85864.12*** | 1082.193*** |
| *Error* | 12 | 0.0488 | 0.0078 | 0.0182 | 9.16 | 2.254 | 2.254 |

EMR(%), emergence; T_50_ (days), Number of days to reach 50% of the final germination; EV (1-9), Early vigor; DS, Days to silking; DA, Days to anthesis; ASI, Anthesis silking interval; NLP, Number of leaves per plant; NEP, Number of ears per plant; EH (cm), Ear Height; PLH (cm), Plant height; ERN, Ear Row Number; NKR, number of kernel per row; EL (cm),Ear length; ED (cm), Ear Diameter; CD (cm), Cob diameter; RD (cm),Rachis diameter; KL (cm), Kernel length; KW (cm), kernel width; KT (cm), kernel thickness; K%, kernel proportion; EW (kg), Weight of 10 ears; 1000KW (g), 1000 kernel weight; HMC(%), moisture content at harvest; KYP (Mg ha^-1^), Kernel yield per plot.
