# Supplementary material for: Genetic variation and structure of maize populations from Saoura and Gourara oasis in Algerian Sahara
Source: BMC Genet. 2018 Aug 1;19:51. doi: 10.1186/s12863-018-0655-2 (PMC6090932; doi:10.1186/s12863-018-0655-2)
Supplement: Supplementary file 3 — Table S6. Eigenvalues, variances and coefficients associated with first three principal components. (DOCX 15 kb) [file 12863_2018_655_MOESM3_ESM.docx]

**Table S6.** Eigenvalues, variances and coefficients associated with first three principal components.

|  | PC1 | PC2 | PC3 |
| --- | --- | --- | --- |
| Eigenvalue | 10.630 | 2.724 | 2.244 |
| Variance (%) | 44.293 | 11.350 | 9.351 |
| Cumulative variance(%) | 44.293 | 55.643 | 64.995 |
|  |  |  |  |
|  | **Coefficientvector** | | |
| EMR | -0.120 | -0.208 | 0.488 |
| T50 | 0.469 | -0.439 | -0.231 |
| E.V | 0.056 | 0.079 | **0.817** |
| DS | **0.869** | -0.263 | 0.036 |
| DA ) | **0.887** | -0.210 | -0.102 |
| ASI | -0.200 | -0.211 | **0.613** |
| NL | 0.577 | 0.028 | -0.161 |
| NEP | -0.051 | **-0.568** | -0.132 |
| PLH | **0.886** | -0.214 | -0.086 |
| EH | **0.849** | -0.269 | -0.037 |
| ERN | **0.847** | -0.024 | -0.223 |
| NKR | **0.899** | -0.034 | 0.122 |
| EL | **0.850** | -0.161 | 0.150 |
| ED | **0.903** | 0.218 | 0.174 |
| CD | **0.828** | 0.246 | -0.078 |
| RD | **0.861** | 0.206 | -0.073 |
| KL | **0.737** | 0.343 | 0.233 |
| KW | -0.057 | **0.591** | 0.471 |
| KT | -0.274 | **0.570** | -0.098 |
| K% | -0.245 | -0.437 | **0.531** |
| EW | **0.956** | 0.147 | 0.159 |
| 1000 KW | 0.254 | **0.745** | -0.173 |
| HMC | 0.091 | -0.366 | 0.035 |
| KYP | **0.897** | 0.071 | 0.297 |
