# Supplementary figures and images for: Genetic variation and structure of maize populations from Saoura and Gourara oasis in Algerian Sahara
Source: BMC Genet. 2018 Aug 1;19:51. doi: 10.1186/s12863-018-0655-2 (PMC6090932; doi:10.1186/s12863-018-0655-2)

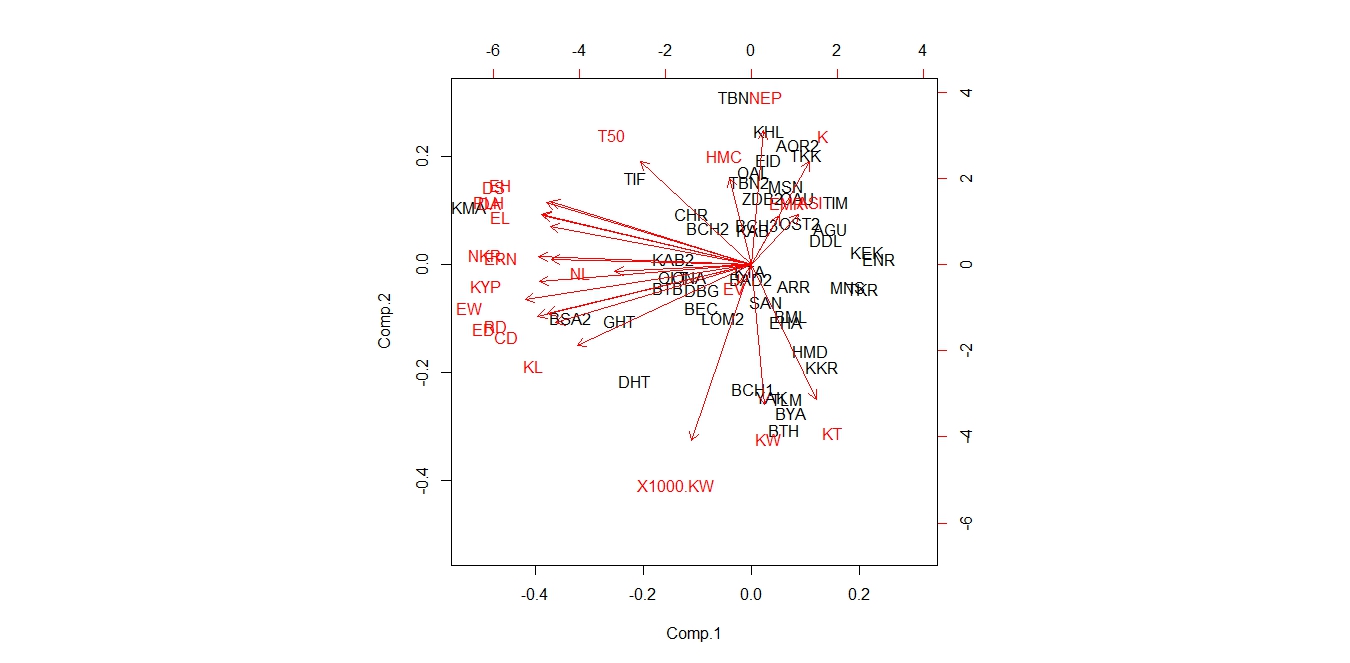

Supplement: Supplementary file 4 — Figure S1. Principal component analysis of the 47 Algerian maize landraces based on 24 agro-morphological traits. (JPEG 152 kb) [file 12863_2018_655_MOESM4_ESM.jpeg]
