# Supplementary material for: Genetic variation and structure of maize populations from Saoura and Gourara oasis in Algerian Sahara
Source: BMC Genet. 2018 Aug 1;19:51. doi: 10.1186/s12863-018-0655-2 (PMC6090932; doi:10.1186/s12863-018-0655-2)
Supplement: Supplementary file 5 — Table S7. Landraces summary statistics based on SSR analysis. (DOCX 14 kb) [file 12863_2018_655_MOESM5_ESM.docx]

**Table S7.** Landraces summary statistics based on SSR analysis.

| **Landrace** | **Na** | **H** | **No. unique alleles** |
| --- | --- | --- | --- |
| *AUG* | 4.33 | 0.59 | - |
| *AOR2* | 3.53 | 0.53 | - |
| *ARR* | 3.44 | 0.51 | - |
| *BAD2* | 3.65 | 0.49 | - |
| *BCH2* | 3.81 | 0.50 | - |
| *BCH3* | 4.17 | 0.52 | - |
| *BEC* | 4.35 | 0.53 | - |
| *BML* | 3.44 | 0.46 | 1 |
| *BSA2* | 4.05 | 0.52 | - |
| *BTB* | 3.5 | 0.44 | 2 |
| *BTH* | 3.59 | 0.45 | - |
| *BYA* | 4.47 | 0.56 | - |
| *CHR* | 3.29 | 0.45 | - |
| *DBG* | 4.06 | 0.55 | 2 |
| *DDL* | 4.23 | 0.53 | 1 |
| *DHT* | 3.59 | 0.52 | 3 |
| *EHA* | 3.76 | 0.48 | - |
| *EID* | 2.62 | 0.38 | 1 |
| *ENR* | 3.88 | 0.55 | - |
| *GHT* | 2.89 | 0.43 | - |
| *HMD* | 4.41 | 0.59 | 2 |
| *KAB2* | 3.41 | 0.49 | - |
| *KAB* | 3.94 | 0.51 | - |
| *KEK* | 3.61 | 0.48 | - |
| *KHL* | 3.18 | 0.42 | - |
| *KKR* | 4.11 | 0.49 | - |
| *KMA* | 3.44 | 0.41 | - |
| *KTA* | 3.44 | 0.49 | 1 |
| *LOM2* | 3.72 | 0.46 | 1 |
| *MNS* | 4.05 | 0.49 | - |
| *MSN* | 4.12 | 0.55 | 1 |
| *OAL* | 3.59 | 0.42 | 1 |
| *OAU* | 3.54 | 0.42 | - |
| *OLT* | 3.55 | 0.45 | - |
| *ONA* | 3.70 | 0.51 | - |
| *OST2* | 3.44 | 0.42 | - |
| *SAN* | 3.94 | 0.52 | - |
| *TBN2* | 3.5 | 0.46 |  |
| *TBN* | 4.05 | 0.55 | - |
| *TIF* | 4 | 0.48 | - |
| *TIM* | 4.28 | 0.52 | - |
| *TKK* | 4.44 | 0.56 | 1 |
| *TKR* | 3.41 | 0.47 | - |
| *TLM* | 3.88 | 0.53 | - |
| *YAK* | 4.53 | 0.59 | 1 |
| *BCH1* | 3.28 | 0.46 | - |
| *ZDB2* | 2.94 | 0.41 | - |

Na, average number of alleles; H, gene diversity.
