# Supplementary material for: Genetic variation and structure of maize populations from Saoura and Gourara oasis in Algerian Sahara
Source: BMC Genet. 2018 Aug 1;19:51. doi: 10.1186/s12863-018-0655-2 (PMC6090932; doi:10.1186/s12863-018-0655-2)
Supplement: Supplementary file 6 — Table S8. Analysis of Molecular Variance. (DOCX 12 kb) [file 12863_2018_655_MOESM6_ESM.docx]

**Table S8.** Analysis of Molecular Variance

| **Source** | **d.f.** | **Sum of Square** | **Mean of Square** | **Variance components.** | **% of variation** |
| --- | --- | --- | --- | --- | --- |
| ***Among populations*** | 46 | 2790.753 | 60.669 | 1.881 | 30% |
| ***Within populations*** |  |  |  |  |  |
| *Among Individuals* | 658 | 2792.233 | 4.244 | 0.000 | 1% |
| *Within Individuals* | 705 | 3003.500 | 4.260 | 4.260 | 69% |
| ***Total*** | 1409 | 8586.487 |  | 6.141 | 100% |
