# Supplementary material for: Genetic variation and structure of maize populations from Saoura and Gourara oasis in Algerian Sahara
Source: BMC Genet. 2018 Aug 1;19:51. doi: 10.1186/s12863-018-0655-2 (PMC6090932; doi:10.1186/s12863-018-0655-2)
Supplement: Supplementary file 7 — Table S9. Partition of the gene diversity among clusters. (DOCX 12 kb) [file 12863_2018_655_MOESM7_ESM.docx]

**Table S9.** Partition of the gene diversity among clusters

| Statistics | Overall | Cluster groups | | | | |  |
| --- | --- | --- | --- | --- | --- | --- | --- |
|  |  | A | B | C | D | E | |
| Sample size | 47 | 29 | 14 | 2 | 1 | 1 | |
| No. unique alleles | 18 | 14 | 4 | 0 | 0 | 0 | |
| Gene diversity (H) | 0.46 | 0.477 | 0.431 | 0.46 | 0.415 | 0.25 | |
