# Supplementary material for: Genetic variation and structure of maize populations from Saoura and Gourara oasis in Algerian Sahara
Source: BMC Genet. 2018 Aug 1;19:51. doi: 10.1186/s12863-018-0655-2 (PMC6090932; doi:10.1186/s12863-018-0655-2)
Supplement: Supplementary file 8 — Table S1. List of the 47 Algerian maize landraces. (DOCX 22 kb) [file 12863_2018_655_MOESM8_ESM.docx]

**Table S1.** List of the 47 Algerian maize landraces.

| **Landrace No.** | **Code** | **Collection site** | **District** | **State** | **Kernel color** | **Kernel type** |
| --- | --- | --- | --- | --- | --- | --- |
| **1** | AOR2 | Aougrout | Aougrout | Adrar | O | F |
| **2** | KHL | K'sar El Hadj | Aougrout | Adrar | O | F |
| **3** | TBN2 | Tiberghamine | Aougrout | Adrar | O/B | F |
| **4** | TBN | Tiberghamine | Aougrout | Adrar | O/B | F |
| **5** | HMD | Hammad | Aougrout | Adrar | O/B | F |
| **6** | KAB2 | Kaberten | Aougrout | Adrar | O/B | F |
| **7** | KAB | Kaberten | Aougrout | Adrar | O/B/Y | F/D |
| **8** | OAU | Ouled abou | Deldoul | Adrar | O/W | F |
| **9** | DDL | Deldoul | Deldoul | Adrar | O/B | F |
| **10** | EHA | Sahla | Deldoul | Adrar | O/B | F/SD |
| **11** | MNS | Mansour | Deldoul | Adrar | O/B/W | F |
| **12** | LOM2 | Metarfa | Metarfa | Adrar | O/Y | F/D/SD |
| **13** | OAL | Ouled Ali | Metarfa | Adrar | O/Y | F |
| **14** | ARR | Amerrade | Charouine | Adrar | O/W | F |
| **15** | BML | Bni Mehlale | Charouine | Adrar | O/Y | F/SD |
| **16** | CHR | Charouine | Charouine | Adrar | O/Y | F |
| **17** | SAN | Sane | Charouine | Adrar | O/R/Y | F/D |
| **18** | AGU | Ain Glou | Talmine | Adrar | O/B/Y | F |
| **19** | ENR | El Nerma | Talmine | Adrar | O | F |
| **20** | TLM | Talmine | Talmine | Adrar | O | F |
| **21** | GHT | Tamest, Gharmianou | Fenoughil | Adrar | O | F |
| **22** | BYA | Bouyahia | Timimoun | Adrar | O/B | F |
| **23** | DBG | Dbagh | Timimoun | Adrar | O/Y/B | F |
| **24** | KEK | K'sar El Kaf | Timimoun | Adrar | O/Y/B | F |
| **25** | KMA | K'sar mainon | Timimoun | Adrar | O/Y | D/SD/F |
| **26** | KTA | K'sar Tmana | Timimoun | Adrar | O/Y/B | F/D/SD |
| **27** | MSN | Massine | Timimoun | Adrar | O/Y/B | F |
| **28** | OLT | Ouahrout | Timimoun | Adrar | O/Y | F/SD |
| **29** | OST2 | Ouled Said | Timimoun | Adrar | Y/B/P | F |
| **30** | TIM | Timimoun | Timimoun | Adrar | O | F/SD |
| **31** | TKR | Tela | Timimoun | Adrar | O | F |
| **32** | YAK | Yakourt | Timimoun | Adrar | O/B | F |
| **33** | TKK | Tinnerkouk | Tinnerkouk | Adrar | O/B/Y | F |
| **34** | ZDB2 | Zaouiet Debagh | Tinnerkouk | Adrar | O/Y/W | F/SD |
| **35** | KKR | K’sar Kaddour | Tinerkouk | Adrar | O/Y/B | F |
| **36** | BAD2 | Badrian | Tsabit | Adrar | O/B/Y | F/SD |
| **37** | BTH | Bentalha | Tsabit | Adrar | O/B | F/D/SD |
| **38** | EID | El Haiad | Tsabit | Adrar | O/Y | F/SD |
| **39** | BCH1 | Bechar | Bechar | Bechar | O | F/SD |
| **40** | BCH2 | Béni Abbes | Béni Abbes | Bechar | O/Y/B | F/SD/D |
| **41** | BCH3 | Igli | Igli | Bechar | O/Y | F/SD |
| **42** | BSA2 | Si Beliane | Lahmer | Bechar | O/B | F/D/SD |
| **43** | ONA | Ouina (Kenadsa) | Ouina (Kenadsa) | Bechar | O/B | F/SD |
| **44** | BTB | Berahi | Taghit | Bechar | O/Y | F/D |
| **45** | BEC | Gherdaïa | Gherdaïa | Gherdaïa | W/Y/O | F/SD |
| **46** | DHT | Doui Thabet | Doui Thabet | Saida | O/B | F |
| **47** | TIF | Tifrit | Tifrit | Saida | O/R/B/Y | F/SD |

Kernel color: O Orange; B Brown; Y Yellow; W White; R Red

Kernel type: F Flint; DDent; SD Semi-Dent
