# Supplementary material for: Genetic variation and structure of maize populations from Saoura and Gourara oasis in Algerian Sahara
Source: BMC Genet. 2018 Aug 1;19:51. doi: 10.1186/s12863-018-0655-2 (PMC6090932; doi:10.1186/s12863-018-0655-2)
Supplement: Supplementary file 9 — Table S2. Agro-morphological traits recorded in the 47 Algerian maize landraces. (DOCX 15 kb) [file 12863_2018_655_MOESM9_ESM.docx]

**Table S2.** Agro-morphological traits recorded in the 47 Algerian maize landraces

| **Abreviation** | **Trait description** | | | | | |  | **Units** |
| --- | --- | --- | --- | --- | --- | --- | --- | --- |
| ***EMR*** | *Emergence proportion of plantlets emergence* |  |  |  |  | |  | % |
| ***T50*** | *Number of days to reach 50% of the finale % of emergence* | | | | | |  | days |
| ***EV*** | *Early vigor(1-9: 1 = weak to 9 = vigourous)* | | | |  | |  | 1-9 |
| ***DS*** | *Days to silking: from planting to when silks have emerged on 50% of the plants* | | | | | |  | days |
| ***DA*** | *Days to anthesis: from planting to when 50% of the plants have shed pollen* | | | | | |  | days |
| ***ASI*** | *Anthesis silking interval* | |  |  |  | |  | days |
| ***NLP*** | *Number of leaves per plant* |  |  |  |  | |  | No. |
| **NEP** | *Number of ears per plant* | |  |  |  | |  | No. |
| **PLH** | *Plant height* |  |  |  |  | |  | cm |
| **EH** | *Ear height* |  |  |  |  | |  | cm |
| **ERN** | *Ear row number* |  |  |  |  | |  | No. |
| **NKR** | *Number of kernels per row* | |  |  |  | |  | No. |
| ***EL*** | *Ear length* |  |  |  |  | |  | cm |
| **ED** | *Ear diameter* |  |  |  |  | |  | cm |
| **CD** | *Cob diameter* |  |  |  |  | |  | cm |
| **RD** | *Rachis diameter* |  |  |  |  | |  | cm |
| **KL** | *Kernel length* |  |  |  |  |  | | cm |
| **KW** | *Kernel width* |  |  |  |  |  | | cm |
| **KT** | *Kernelthickness* |  |  |  |  |  | | cm |
| **K%** | *Kernel proportion* |  |  |  |  | |  | % |
| ***EW*** | *Weight of 10 ears* |  |  |  |  | |  | kg |
| **1000 KW** | *1000 kernel weight* | |  |  |  | |  | g |
| **HMC** | *Moisture content at harvest* | |  |  |  | |  | % |
| **KYP** | *Kernel yeild per plot* | |  |  |  | |  | Mgha^-1^ |
