# Supplementary material for: Genetic variation and structure of maize populations from Saoura and Gourara oasis in Algerian Sahara
Source: BMC Genet. 2018 Aug 1;19:51. doi: 10.1186/s12863-018-0655-2 (PMC6090932; doi:10.1186/s12863-018-0655-2)
Supplement: Supplementary file 10 — Table S3. Primers used to detect SSR marker. (DOCX 14 kb) [file 12863_2018_655_MOESM10_ESM.docx]

**Table S3.** Primers used to detect SSR marker

| ***Marker*** | ***Bin*** | ***Motif*** | ***Forward*** | ***Reverse*** |  |  |
| --- | --- | --- | --- | --- | --- | --- |
| ***umc1222*** | 1.01 | (AG)20 | CTCAGAACAGAAGCCATCAAAAGC | CGTCTTCGTGAGAGACATCCTGT | | |
| ***umc1403*** | 1.03 | (GCA)4 | GTACAACGGAGGCATTCTCAAGTT | TGTACATGGTGGTCTTGTTGAGGT | | |
| ***umc1335*** | 1.06 | (AG)24 | ATGGCATGCATGTGTTTGTTTTAC | ACAGACGTCGCTAATTCCTGAAAG | | |
| ***umc1165*** | 2.01 | (TA)6 | TATCTTCAGACCCAAACATCGTCC | GTCGATTGATTTCCCGATGTTAAA | | |
| ***umc1265*** | 2.02 | (TCAC)4 | GCCTAGTCGCCTACCCTACCAAT | TGTGTTCTTGATTGGGTGAGACAT | | |
| ***phi127*** | 2.08 | AGAC | ATATGCATTGCCTGGAACTGGAAGGA | AATTCAAACACGCCTCCCGAGTGT | | |
| ***bnlg1520*** | 2.09 | (AG)22 | TCCTCTTGCTCTCCATGTCC | ACAGCTGCGTAGCTTCTTCC | | |
| ***phi036*** | 3.04 | (AG)n | CCGTGGAGAGACGTTTGACGT | TCCATCACCACTCAGAATGTCAGTGA | | |
| ***umc1963*** | 4.04 | (AGC)3 | CTCGTTCGAGGGGATGTACAAG | CTTGCACTGGCACAGAGACG | | |
| ***umc1329*** | 4.06 | (GCC)7 | CCTCTCACATCTCCTCTCCCCT | GTGTCGGTGTAGGTCTCCGTCTT | | |
| ***umc1225*** | 5.08 | (AG)6 | CTAGCTCCGTGTGAGTGAGTGAGT | TTCCTTCTTTCTTTCCTGTGCAAC | | |
| ***umc1424*** | 6.06 | (TCC)7 | CCGGCTGCAGGGGTAGTAGTAG | ATGGTCAGGGGCTACGAGGAG | | |
| ***bnlg1740*** | 6.07 | (AG)21 | TTTTCTCCTTGAGTTCGTTCG | ACAGGCAGAGCTCTCACACA | | |
| ***umc1545*** | 7.00 | (AAGA)4 | GAAAACTGCATCAACAACAAGCTG | ATTGGTTGGTTCTTGCTTCCATTA | | |
| ***umc1327*** | 8.01 | (GCC)4 | AGGGTTTTGCTCTTGGAATCTCTC | GAGGAAGGAGGAGGTCGTATCGT | | |
| ***umc1984*** | 8.03 | (CAG)3 | CTCTGGCCTCTGATACCAGTTGAT | CATCCTCCTGCAGCTGTTAACTC | | |
| ***phi027*** | 9.03 | (GCGCT)n | GCGTACGTACGACGAAGACAC | CACAGCACGTTGCGGATTTCTCT | | |
| ***phi059*** | 10.02 | (ACC)n | AAGCTAATTAAGGCCGGTCATCCC | TCCGTGTACTCGGCGGACTC | | |
